# Human cerebrovascular function in health and disease: insights from integrative approaches

**DOI:** 10.1186/s40101-018-0164-z

**Published:** 2018-02-17

**Authors:** Erin D. Ozturk, Can Ozan Tan

**Affiliations:** 1 Cerebrovascular Research Laboratory, Spaulding Rehabilitation Hospital, Boston, MA USA; 2 Department of Psychology, Harvard University, Cambridge, MA USA; 3 Department of Physical Medicine and Rehabilitation, Harvard Medical School, Boston, MA USA

**Keywords:** Neurovascular coupling, Vasoreactivity, Autoregulation, Cerebral blood flow

## Abstract

**Background:**

The marked increase in the size of the brain, and consequently, in neural processing capability, throughout human evolution is the basis of the higher cognitive function in humans. However, greater neural, and thus information processing capability, comes at a significant metabolic cost; despite its relatively small size, the modern human brain consumes almost a quarter of the glucose and oxygen supply in the human body.

Fortunately, several vascular mechanisms ensure sufficient delivery of glucose and oxygen to the active neural tissue (neurovascular coupling), prompt removal of neural metabolic by-products (cerebral vasoreactivity), and constant global blood supply despite daily variations in perfusion pressure (cerebral autoregulation). The aim of this review is to provide an integrated overview of the available data on these vascular mechanisms and their underlying physiology. We also briefly review modern experimental approaches to assess these mechanisms in humans, and further highlight the importance of these mechanisms for humans’ evolutionary success by providing examples of their healthy adaptations as well as pathophysiological alterations.

**Conclusions:**

Data reviewed in this paper demonstrate the importance of the cerebrovascular function to support humans’ unique ability to form new and different interactions with each other and their surroundings. This highlights that there is much insight into the neural and cognitive functions that could be gleaned from interrogating the cerebrovascular function.

## Background

Among the most salient features of human evolution is the dramatic increase in the brain size from early hominins (e.g., genus *Ardipithecus*) to *Homo sapiens*. This increase marks the basis of the higher cognitive function attributed to humans, such as new and different interactions with each other (e.g., social interactions) and their surroundings (e.g., tool use), and underlies their ability to face unfamiliar habitats. This level of the cognitive function is uniquely “human”; compared to their primate relatives, modern humans have far more white matter in their prefrontal cortex [1, 2], reflecting denser neural connectivity, and consequently, a greater ability to process information.

However, greater neural, and thus information processing capability, comes at a significant metabolic cost. Despite its relatively small size compared to other organs, the modern human brain consumes almost one quarter of the glucose and oxygen supply in the human body [3, 4]. The constraints imposed on the neural function by this demand are further compounded by the fact that neural metabolism is almost entirely aerobic and that neurons do not store enough glucose to function on their own [3]. That is, neural cells rely on external glucose and oxygen supply for metabolic activity (contrast this to, e.g., muscle cells, which can briefly utilize carbohydrates in the absence of sufficient oxygen for anaerobic metabolism). Therefore, there is a constant need for glucose and oxygen supply to the brain for sufficient neural function. However, the evolution of brain size and higher cognitive function was also coupled with the evolution of bipedal movement, resulting in yet another significant constraint to the blood, thus, glucose and oxygen supply to the brain: the upright posture and consequent gravitational force against the blood flow above the heart level. Fortunately, several physiologic mechanisms ensure sufficient delivery of glucose and oxygen to active neural tissue, prompt removal of neural metabolic by-products, and constant global blood supply despite gravitational forces and daily variations in perfusion pressure.

Study of cerebrovascular circulation dates back to the mid-nineteenth century, when Donders observed that asphyxia causes dilation of cerebral blood vessels [5]. Near the turn of the century, Roy and Sherrington argued that vasoconstrictor nerves cause constriction in response to anoxia, but that metabolic bi-products lead to dilation of cerebral blood vessels [6]. However, Hill argued that cerebral blood flow was an entirely passive function of changes in blood pressure [7], a perspective that dominated physiologic inquiry for quite some time. By the middle of the twentieth century, it was recognized that there was evidence for increased blood flow to areas of increased brain activity, but that total blood supply remained remarkably constant [8, 9]. To assess the underlying physiological mechanisms of this maintenance in flow, Guyton and associates [10, 11], in technically difficult physiological experiments yet to be replicated, isolated the cerebral circulation of one dog from his peripheral circulation, by supplying it from another, donor dog. The carotid sinus nerves of the recipient dog were cut, eliminating baroreceptor and chemoreceptor responses to pressure changes. Under these circumstances, the recipient dog showed no signs of maintenance of flow, but instead cerebral blood flow simply tracked arterial blood pressure over a range of 20 to 140 mmHg. These studies established the groundwork for the modern research to understand regulation of cerebral blood flow.

In this paper, we will first review our current understanding of the components of cerebrovascular control and the physiology that underlies them. Next, we will provide a brief review of modern experimental approaches to assess these components in humans. An understanding of the strengths and weaknesses of these approaches is necessary to better interpret the findings of studies assessing the cerebrovascular function, the focus of this review. Subsequently, we will highlight the importance of the cerebrovascular function for humans’ evolutionary success by providing an overview of healthy adaptations as well as pathophysiological alterations of the cerebrovascular function and their consequences.

## Physiology of cerebrovascular control

The brain seems to lack the survival advantage of other organs that are more tolerant to fluctuations in blood flow due to its dependence on aerobic metabolism and constraints imposed by bipedal posture on blood supply. However, a large number of studies have shown that this disadvantage is compensated by three mechanisms to ensure that brain perfusion is maintained and regulated appropriately: neurovascular coupling, cerebral vasoreactivity, and cerebral autoregulation.

### Neurovascular coupling

The distribution of cerebral flow is regulated according to the functional activity of different brain regions. This link between increased metabolic demand and increased blood flow is termed *neurovascular coupling*. Alterations in this mechanism can impair the ability of the brain vasculature to provide sufficient flow to active regions, leading to neural dysfunction [12].

It is generally thought that regional cerebral blood flow and cerebral metabolic rate are normally coupled because neuronal activity requires delivery of adequate glucose and oxygen to specific brain regions [12]. However, it should be noted that the increase in regional cerebral blood flow in response to local neural brain activation is not altered by mild to moderate hypoglycemia (low blood glucose concentration) [13], and regional cerebral blood flow does not appear to be affected by elevated arterial oxygen concentration neither at rest nor in response to somatosensory stimulation [14]. Moreover, regional responses of cerebral blood flow to physiologic or pathophysiologic neuronal activation appears to be independent of the level of oxygen transported to the tissue [15]. Thus, the lack of glucose alone does not fully explain regional blood flow responses, and a shortage of oxygen may not be among the primary drivers of vasodilation (hence, the increase in regional blood flow) during increased neuronal activity.

Instead, vascular responses to neural activation appear to be tightly controlled by the afferent inputs to the activated region, and astroglial signals appear to be the primary effectors in conveying integrated neural signals and neuronal activity into a vascular response [16]. Astrocytes directly contact endothelial cells on the vascular smooth muscle and release a number of vasodilatory substances, such as nitric oxide [17]. While the vasodilatory effects in the local microcirculation alone may be insufficient to effectively increase local blood flow, the vasodilatory signal are propagated back to upstream pial arterioles via gap junctions of neighboring endothelial cells. This is necessary as pial arterioles offer the greatest resistance to blood flow. Thus, the vascular endothelial function and smooth muscle responsiveness appear to be critical in transducing the signals so that cerebral vasculature acts in concert to increase regional cerebral flow to support neural activation.

### Cerebral vasoreactivity

A second component of cerebrovascular control, termed *cerebral vasoreactivity*, is the high sensitivity of cerebral vasculature to changes in arterial CO_2_ and oxygen (O_2_) levels. High CO_2_ (hypercapnia) leads to vasodilation and increases flow. In contrast, low CO_2_ (hypocapnia) leads to vasoconstriction and decreases in flow. This highly sensitive flow response is a vital homeostatic function as arterial CO_2_ can fluctuate widely from one breath to the next and can change significantly with everyday postural changes.

This response to arterial CO_2_ appears to be global. For example, in response to a breath-hold maneuver, which increases arterial CO_2_ roughly proportionally to the length of breath-hold (but see the next section for caveats), there is approximately 1.7- to 1.9-fold increase in cerebral blood flow in the middle cerebral, as well as basilar arteries for every second of breath holding, without any apparent lateral differences [18]. Moreover, this response is primarily due to the change in blood acidity: global cerebral blood flow increases in a dose-dependent fashion and for up to 15 min in response to systemic administration of acetazolamide, though the dose dependency does not appear to be the case for regional blood flow responses [19]. Acetazolamide inhibits carbonic anhydrase, the catalyzer of the hydration reaction of carbon dioxide, thereby elevating blood acidity without any change in blood gas concentrations [20]. Thus, vasodilation in response to hypercapnia “washes out” CO_2_ from brain tissue, thereby attenuating the rise in blood pH, and conversely, vasoconstriction in response to hypocapnia attenuates the fall in brain pH.

While the mechanism underlying these CO_2_-mediated blood flow changes has not been entirely elucidated, CO_2_/pH-induced alterations in vasoactive factors, such as endothelial release of nitric oxide (NO), are essential. However, it is important to note that the brain (i.e., neural), and not vascular (i.e., endothelial) NO, may have an important role in the response to hypercapnia, although the latter may have a permissive role in this response [21]. In addition, autonomic sympathetic outflow appears to impact cerebral vasoreactivity. For example, in an elegant study in dogs, Harper and Glass [22] lowered arterial pressure (by controlled hemorrhage), which is expected to elevate sympathetic outflow, and reported that cerebral vasoreactivity was blunted. This is consistent with data from humans; cerebral vasoreactivity is reported to increase by almost 50% during ganglionic (i.e., sympathetic and cholinergic) blockade in humans [23]. Thus, sympathetic outflow, which is primarily vasoconstrictive, may restrain CO_2_-mediated vasodilation. On the other hand, alpha-adrenergic agonist ephedrine [24] or alpha/beta-adrenergic antagonist labetalol [25] do not appear to impact cerebral vasoreactivity. Therefore, the exact mechanisms that underlie cerebral vasoreactivity remain unclear.

### Cerebral autoregulation

The third mechanism—*cerebral autoregulation*—counteracts the fluctuations in systemic arterial pressure that occur in everyday activities. For example, changes in posture can result in as much as a 50% drop in systolic pressure and produce vasovagal syncope with brief loss of consciousness, if blood flow to reticular brain cells falls rapidly [6]. Cerebral circulation is a high-flow vascular bed encased in a non-distensible skull; inadequate blood flow leads to brain damage and neural degeneration, and increased perfusion leads to increased intracranial pressure that can also lead to neural degeneration and cell death due to blood vessel and tissue compression. Fortunately, cerebral vasculature is able to regulate perfusion in response to hypotension and to swings in arterial pressure [26–28]. This mechanism—autoregulation—ensures that transient fluctuations in pressure (e.g., due to respiration) are transmitted to the cerebral circulation almost linearly, whereas slower fluctuations that may result in greater sustained impact on neurophysiological health (by causing prolonged changes in cerebral perfusion) are effectively buffered against. Thus, intact cerebral autoregulation is critical to neurophysiologic health.

Original observations of cerebral flow responses [29] supported a counter-regulation against changes in arterial pressure encompassing the time scale from minutes to hours. Effective autoregulation results in maintained cerebral blood flow via cerebrovascular resistance changes that fully counteract changes in arterial pressure [30, 31]. In the modern literature, a distinction has been made between this “static” and “dynamic” autoregulation; the latter takes place over several seconds or beats. This is based on recent work showing that there is a close linear relation between changes in arterial pressure and cerebral blood flow that occur almost synchronously when arterial pressure fluctuations are relatively fast (faster than ~ 10 s), and that this linear relation gradually disappears as fluctuations become slower [27, 28, 32]. However, there may be no compelling physiological or pragmatic evidence to indicate that static and dynamic autoregulation are distinctly different mechanisms [32].

There are strong inferential data suggesting that the autonomic (sympathetic and cholinergic) nervous system plays a role in cerebrovascular regulation. For example, the magnitude of spontaneous fluctuations in flow in relation to those in pressure is related to the severity of carotid stenosis [33], which can markedly impair autonomic control [34]. In addition, in response to acute sympathoexcitatory stimuli (e.g., isometric exercise [35], simulated orthostatic stress [36], cold pressor test [37]), cerebrovascular resistance increases. Moreover, after ganglionic blockade with trimethaphan [38], or systemic pharmacologic blockade of alpha-adrenergic [39] or muscarinic cholinergic receptors [40], the gain (i.e., transfer function magnitude) between cerebral flow and systemic pressure almost doubles, indicating that the degree of cerebral counter-regulation to pressure fluctuations was reduced. These data are strongly suggestive of an autonomic role in cerebral blood flow control. In fact, more recently, we have shown that in humans, autonomic sympathetic and cholinergic control is responsible for homeostatic maintenance of cerebral blood flow in response to arterial pressure fluctuations within physiologic range, and local myogenic (i.e., calcium channel-mediated) control may be neuroprotective against ischemia and hemorrhage when swings in pressure are rapid and large [41]. Therefore, intact autonomic function is critical to normal cerebrovascular responses to changes in arterial pressure.

### Interactions between the components of the cerebrovascular function

It is important to note that individual components of the cerebrovascular function do not act in isolation, but interact to ensure proper neural perfusion. For example, we have recently shown that fluctuations in CO_2_ along with those in cardiac output determine the magnitude of slow fluctuations in cerebral blood flow and that cerebrovascular responses to arterial blood pressure fluctuations become pronounced only when central volume shift is pronounced [42]. In addition, recent data show that in healthy volunteers, engagement of cerebral autoregulation appears to blunt neurovascular coupling [43]. Thus, there is a significant interaction between the mechanisms that underlie neurovascular coupling, autoregulation, and vasoreactivity. Changes in mean arterial pressure and those in partial pressure of CO_2_ demonstrate a strong positive correlation only during ganglionic blockade in humans [23], and in those with autonomic dysfunction, visually evoked increases in cerebral blood flow are reduced [44] and cognitive performance is somewhat reduced [45, 46] during orthostatic stress. These data are highly suggestive of autonomic control as the mediator of the interaction between components of the cerebrovascular function. Nonetheless, specifics of these interactions and physiology that underlies them remain largely unknown.

## Experimental approaches to assess the cerebrovascular function in humans: a brief overview

To measure cerebral blood flow, earlier studies of autoregulation relied on inert gas and dilution methods which are limited by both a poor time resolution and, in some cases, by very few observations [47]. Because of these limitations, earlier studies of the cerebrovascular function actually pooled data across subjects. Despite evident limitations of such techniques, this work did shed light on cerebral blood flow regulation and lay the groundwork for more recent work exploiting real-time measurements of pressure and flow.

The recent availability of instrumentation with high temporal resolution, such as finger photoplethysmography coupled with transcranial Doppler ultrasound imaging in the 1980s, allowed utilization of rapid, beat-by-beat measurements of arterial pressure and cerebral blood flow to explore cerebrovascular function cerebral autoregulation within individuals. Assuming that, at least in healthy individuals, cerebrospinal fluid pressure is roughly constant [48], the pressure difference between the cerebral arteries and veins (i.e., intracranial pressure) drives cerebral blood flow, and the pressure in cerebral and peripheral veins is usually very close to the atmospheric pressure [49]. Thus, photoplethysmographic arterial pressure at the level of the head (e.g., at the finger in the supine position) is usually adequate to represent cerebral perfusion pressure. Using transcranial Doppler ultrasonography (TCD), cerebral blood flow velocity can be measured at one of the major cerebral arteries (anterior cerebral, posterior cerebral, middle cerebral, or vertebral artery), although middle cerebral artery (MCA) is most common in the literature. It should be noted that the recorded signal is velocity and not flow. Fortunately, at least for the MCA in healthy individuals, the diameter remains relatively constant despite hypercapnic [50, 51], hypocapnic [50, 52], and orthostatic [53] challenges within physiological range. While acute physical activity and exercise may impact MCA diameter, the extent of the change in MCA does not appear to be more than 2% [54, 55]. Thus, in general, flow velocity can be used as an adequate surrogate for cerebral flow. Once adequate signals are obtained, one can use appropriate stimuli to engage neurovascular coupling, vasoreactivity, and/or autoregulation to assess the components of the cerebrovascular function.

### Neurovascular coupling

The link between increased metabolic demand and increased blood flow—neurovascular coupling—in humans has commonly been explored using functional magnetic resonance imaging (fMRI). However, this may not be always feasible due to the high cost and logistical issues associated with MR imaging, and TCD has been utilized as an alternative measurement of global cerebral blood flow responses to neural activity. It should be noted, however, that spatial and temporal resolution of fMRI and TCD are completely different. Moreover, fMRI and TCD rely, respectively, on blood oxygenation and blood flow velocity, and there is data suggesting that blood oxygenation (measured by near infrared spectroscopy) and blood flow velocity (measured by TCD) responses to neural activity do not always match [56, 57]. Therefore, observations based on MR imaging (or near infrared spectroscopy) and those based on TCD may not always be directly comparable.

For assessment of neurovascular coupling, it is critical to ensure that the stimulus (in this case, cognitive demand) is comparable across individuals. This is essential if one were to compare cerebrovascular responses across different individuals or populations. Moreover, the intensity of the stimuli should be readily measurable (e.g., reaction time and/or percent correct performance), neural areas engaged should be well known (to ascertain that measurements are made from the artery that supplies the neural regions that the stimuli engages), and the time-course of neural activation should be unambiguously defined (so that blood flow responses can be reliably interpreted *vis-à-vis* metabolic demand). One stimulus that meets these criteria and commonly used in the literature is the “*n*-back” task.

**Fig. 1:**
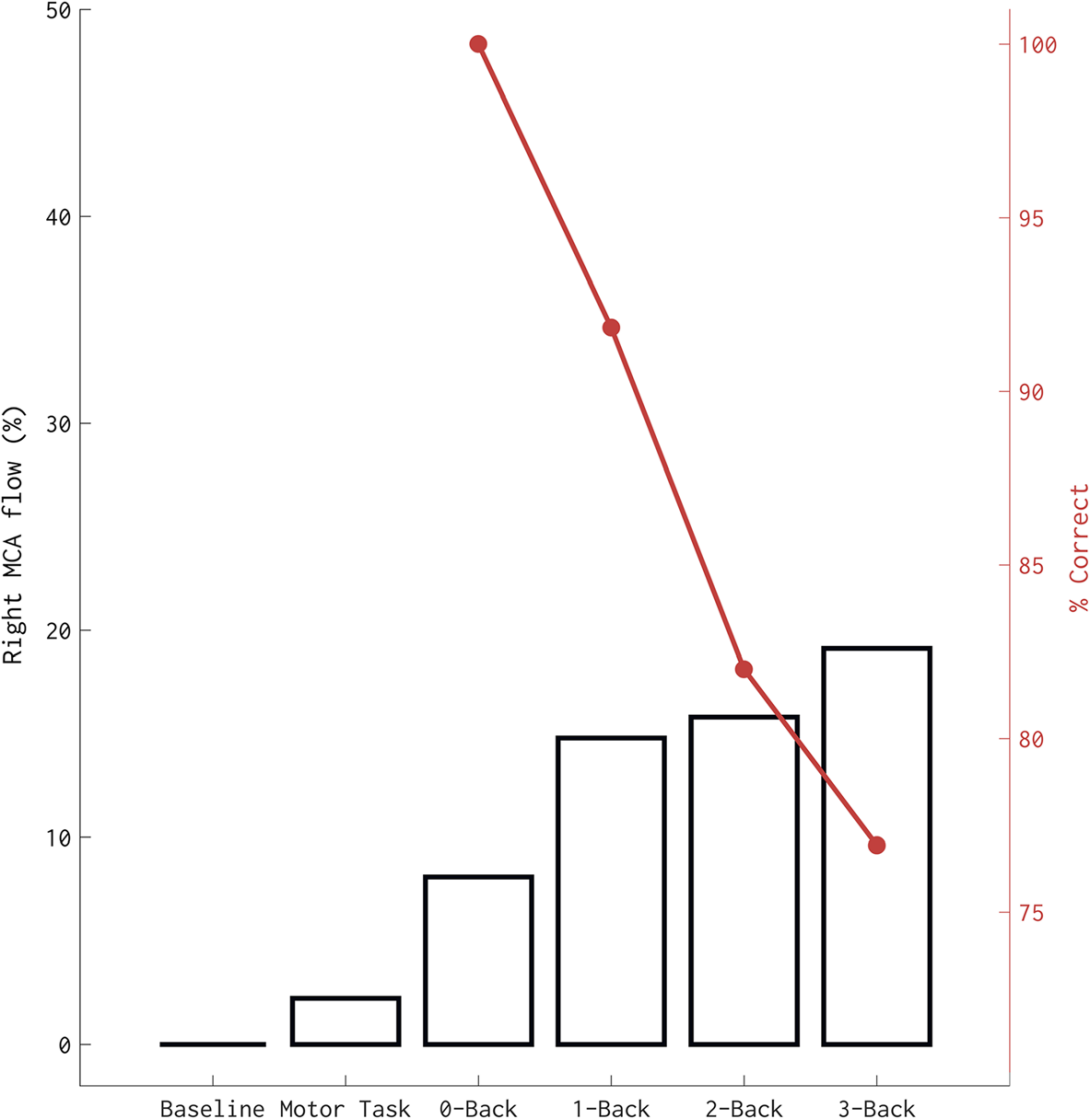
Cerebrovascular responses and task performance (% correct) of one volunteer during baseline, a motor task, an attention task (0-back), and cognitive tasks of increasing difficulty (1-back to 3-back). Note that cerebral blood flow velocity increases in parallel to the increase in neural demand

During the *n*-back task, a series of single letters appear in succession on the screen, and volunteers are asked to click the button each time they see a letter repeated (1-back), each time they see a letter repeated every other letter (2-back), and so on. The number of letters the subject must remember (i.e., the “*n*”) increases until the subject’s performance drops below random chance level (i.e., 50%). This ensures that at the end of the *n*-back task, the cognitive demand is similar and that any difference in cerebral blood flow responses between subjects cannot be attributed to different cognitive efforts. It is also necessary to include two control conditions, especially if neurovascular coupling is assessed across different populations with potentially different attention or motor control abilities (e.g., healthy controls vs individuals with mild cognitive impairment, or individuals with spinal cord injury). An “identify the letter” task, wherein volunteers are asked to click on a button each time they see a letter, can be used to control for cerebral blood flow responses to attentional demand. A motor task, e.g., a sequential finger movement task, can be used to control for cerebral blood flow responses to motor commands. This way, cerebral blood flow responses to neural activity and consequent metabolic demand, i.e., neurovascular coupling can be reliably assessed (Fig. 1).

### Cerebral vasoreactivity

Cerebrovascular responses to arterial CO_2_—cerebral vasoreactivity—is a standard test of cerebrovascular vasodilatory responses. This is typically accomplished by altering arterial CO_2_ concentration by an appropriate maneuver: breath-holding, step changes in CO_2_ (CO_2_ rebreathing to induce hypercapnia and hyperventilation to induce hypocapnia), and progressive increases in CO_2_ induced by air rebreathing.

While breath-holding is perhaps the simplest approach to elicit hypercapnia, it also induces a number of other hemodynamic responses—called the diving reflex—including a reduction in heart rate (bradycardia), a marked reduction in cardiac output, and an increase in arterial blood pressure [58], in addition to acute elevations in sympathetic neural outflow. These responses are partly due to increased intrathoracic pressure. Note that any of these hemodynamic changes can alter cerebral blood flow responses independent of, or at least in addition to arterial CO_2_ (see also above) [38, 39, 41, 42]. In fact, there is evidence that intrathoracic pressure changes alone can alter cerebral blood flow and oxygenation in a way that is similar to the changes associated with breath holding [59]. Therefore, cerebrovascular responses to breath-hold maneuver do not always reflect responses to CO_2_ and should be interpreted carefully.

**Fig. 2:**
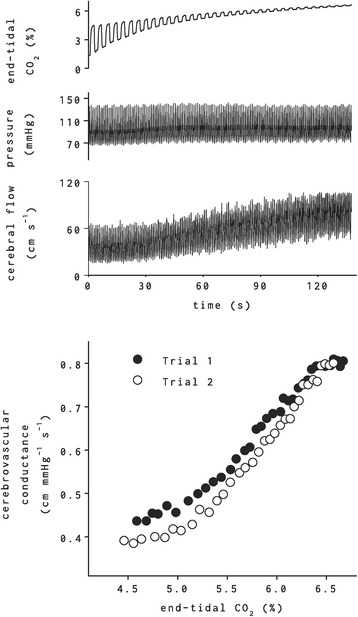
Systemic arterial blood pressure and cerebral blood flow velocity responses to air rebreathing. Note the increase in cerebral blood flow velocity (third panel) in response to increasing end-tidal CO_2_ (first panel) without any apparent increase in arterial blood pressure. Bottom panel shows the change in cerebrovascular conductance (i.e., flow/pressure; to account for any change in pressure, which can alter cerebral blood flow independently) during two separate trials of air rebreathing by the same subject

Two alternative approaches are CO_2_ or air rebreathing. Both approaches essentially increase the level of CO_2_. However, the latter has the advantage of allowing assessment of vascular responses to gradual changes in CO_2_. During air rebreathing, volunteers rebreathe air from a ~ 5-l rebreathing bag until the end-tidal CO_2_ concentration reaches to ~ 6–7% (or ~ 50 mmHg), which typically takes roughly ~ 2 min. Continuous end-tidal CO_2_ levels, measured by a gas analyzer through a sampling tube on the expired side, can then be used to assess breath-by-breath cerebral flow responses (Fig. 2).

### Cerebral autoregulation

In the literature, it is somewhat common to assess cerebral blood flow responses to changes in arterial blood pressure—cerebral autoregulation—by observing the relation between spontaneous blood pressure changes and cerebral blood flow at rest. However, it is important to note that spontaneous pressure fluctuations can be inconsistent due to their small amplitude [60]. As a result, while the observed spontaneous pressure–cerebral blood flow relationship entails periods of low correlation where fluctuations in blood flow may appear with no apparent arterial pressure drive [61], it is not possible to ascertain whether this low correlation is indicative of cerebrovascular counter-regulation or randomness (i.e., noise). To examine the relationship between two signals accurately, one needs fluctuations with sufficient amplitude, often absent in resting steady-state data.

Among the methods commonly used to generate sufficiently large fluctuations is lower body pressure (LBP). Standard application of negative pressure effectively distends the veins in the lower body, causing a caudal shift in blood volume proportional to the level of LBP. This allows study of cardiovascular responses to central blood volume shifts similar to that which occurs during standing, but in a controlled and graded manner without accompanying muscle contraction. While these fluctuations are not greater than those that occur during everyday activities, both negative [27, 28, 32, 42, 43] and positive [42, 62] LBP have been used successfully to probe cerebral autoregulation. As an alternative, several studies used deflation of thigh cuffs to elicit transient blood pressure changes. However, while both the thigh-cuff maneuver and LBP elicit transient changes in arterial pressure, the cerebrovascular responses to the former are not always consistent with those predicted from the relationship derived during the latter, and there is a considerable inter-individual variability in cerebrovascular responses that makes simple averaging of observed responses inappropriate [27]. Baroreflex engagement, concomitant with the sudden caudal blood volume shift consequent to release of ischemic thigh-cuffs [63], may explain some of this discrepancy. Although LBP may also engage the baroreflex, this engagement is inconsistent within and across subjects [64], whereas cerebral blood flow responses to LBP are highly consistent [27]. Thus, moderate LBP is a useful technique for augmenting arterial pressure oscillations at distinct frequencies to generate sufficiently large fluctuations and to engage physiological effectors of autoregulation.

**Fig. 3:**
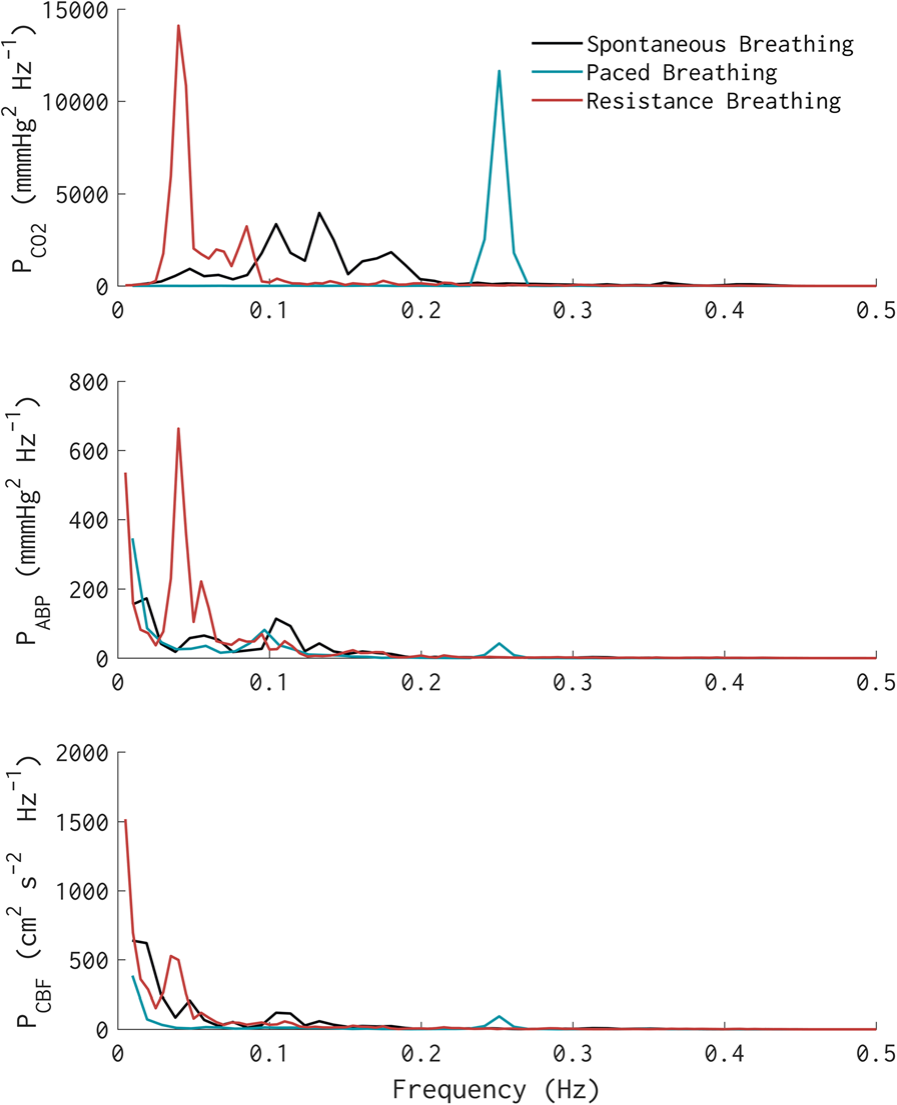
The magnitude of fluctuations (i.e., spectral power) in end-tidal CO_2_ (as a surrogate for respiration; top panel), systemic arterial blood pressure (middle panel), and cerebral blood flow velocity (bottom panel) during spontaneous breathing, paced breathing (at 0.25 Hz; 15 bpm), and resistance breathing (~ 10–20 cmH_2_O resistance, lower panel). Note the marked increase in the magnitude of arterial pressure fluctuations below 0.1 Hz (i.e., slower than 10 s) during resistance breathing, due to increased slow fluctuations in respiration, and thus in intrathoracic pressure. Also note the lack of an increase in cerebral blood flow fluctuations in the same frequency band, indicative of effective cerebral autoregulation

Nonetheless, using LBP in some populations (especially in clinical studies) can be technically very challenging. An alternative approach is to use low resistance breathing to elicit oscillations in arterial blood pressure to characterize the cerebral blood flow response [65]. Volunteers may be asked to breathe through a mouthpiece attached to a standard impedance threshold device set-up to moderate resistance (10–20 cmH_2_O), while their ventilation is closely monitored and coached (so as to avoid hyperventilation, and thus, hypocapnia). Resistance breathing typically requires volunteers to breath deep and slow, providing sufficiently large and consistent arterial pressure fluctuations (due to intrathoracic pressure change as a result of increased tidal volume changes) that are also slow enough to engage cerebral autoregulatory mechanisms (see Fig. 3).

## Healthy adaptations and pathophysiological alterations of human cerebrovascular control

The importance of the cerebrovascular function is perhaps best understood by exploring adaptations of this function in response to changing environmental conditions. For example, exposure to high altitude results in a number of hemodynamic adaptations: sympathetic activity and systemic blood pressure increase [66], and the ventilatory and blood pressure responses to hypoxia are elevated [67]. Each of these systemic changes can impact the cerebrovascular function, and the cerebral vasculature must adapt.

While there is some data suggestive of changes in the cerebrovascular function with high altitude, the nature of this change is equivocal. For example, Willie et al. measured cerebrovascular blood flow patterns during ascent to ~ 5000-m altitude, and in the first 2 weeks of acclimatization to this altitude [68]. They reported that total cerebral blood flow (both internal carotid and vertebral flow and MCA flow) steadily increased by over 50% during the ascent, but gradually returned to ~ 15% of that at the sea level after ~ 2 weeks of acclimatization, without any significant differences in regional cerebral blood flow patterns. Though this may indicate a cerebrovascular adaptation to high altitude, the change in total blood flow was somewhat related to arterial oxygen saturation. This suggests that the alterations in total cerebral blood flow may also be simply due to ventilatory acclimatization. That is, initial increase in global cerebral blood flow may be due to the initial hypoxia, which is gradually offset by reduced partial CO_2_ due to increased ventilation, without any adaptations in the cerebrovascular function. Two studies did report reduced vasoreactivity after 5 [69] and 14 days [70] at high altitude (~ 4300 to 5000 m). However, it is not clear whether this reduction is due to an actual change in cerebrovascular mechanisms or simply due to hypoxia-induced increase in sympathetic nervous outflow [66], which may constrain cerebrovascular vasodilation [22, 23]. On the other hand, Fan et al. [71, 72] reported an elevated cerebral vasoreactivity in response to both CO_2_ and acetazolamide with prolonged exposure to high altitude. It is possible that there may be a reduction in cerebral autoregulatory function due to elevated sympathetic activity, i.e., a more “pressure-passive” relationship between arterial pressure and cerebral blood flow [73]. Indeed, there appears to be proportional increases in arterial pressure and cerebral blood flow, indicative of ineffective autoregulation, among healthy high-altitude natives living at ~ 4200 m [74]. In a more recent study, Fan et al. [75] showed that compared to sea level, cerebral blood flow responses to CO_2_ were exaggerated (by almost 80%) immediately after ascent to ~ 5200 m and were further elevated (by another 90%) after 16 days of acclimatization. However, it is important to note that when cerebrovascular responses were assessed as cerebrovascular conductance (flow over pressure), the latter elevation in responsiveness was no longer evident, suggesting that the apparent increase in cerebrovascular responsiveness during acclimatization may be due to further elevations in arterial pressure, a reduction in the effectiveness of cerebral autoregulatory function, or both. Note that none of these studies explored the impact of high-altitude exposure beyond 2–3 weeks, and limited data suggests that native-born high-altitude residents generally show reduced cerebrovascular reactivity, particularly evident among brain regions that are typically involved in cerebral modulation of respiration [76]. This may indicate that mechanisms that underlie cerebrovascular control can adapt to environmental conditions.

Another example of cerebrovascular adaptations is alterations in response to regular physical activity and fitness. An acute bout of exercise requires integration of the three mechanisms of the cerebrovascular function: cerebral blood flow increases due to sustained muscle engagement, consequent cortical activation, and elevated metabolic activity in motor and sensorimotor areas (cf. neurovascular coupling) [77]. However, this increase in blood flow is not proportional to the intensity of exercise. Blood flow increases up to approximately 60% of the maximum exercise intensity and returns toward baseline at higher intensities [78]. This is due to exercise intensity-dependent levels CO_2_. During an acute bout of aerobic exercise, there is an increase in CO_2_ production that results in vasodilation due to hypercapnia (cf. vasoreactivity) [79]. During high intensity exercise, however, there is a reduction in CO_2_ because ventilation increases exponentially with exercise intensity as pH decreases. Thus, during intense exercise, there is a reduction in cerebral blood flow [80]. Moreover, while there is a progressive increase in arterial blood pressure during exercise, cerebral vasculature appears to be effective in limiting the increase in systolic cerebral blood flow velocity (cf. autoregulation) [81]. Thus, all components of the cerebrovascular function are fully engaged during an acute bout of exercise to ensure appropriate perfusion of neural tissue. Perhaps as a result, frequent physical exercise and better aerobic conditioning are associated with better cerebral blood flow regulation and better cognitive function [82], and conversely, prolonged inactivity results in significant deficits in cerebrovascular control [83]. In addition, there appears to be a modest (*R*^*2*^ ~ 0.20) positive relation between cardiorespiratory fitness and both total and regional cerebral blood flow in the gray matter [84]. This may be related to improved cerebrovascular function. In fact, cerebrovascular response to CO_2_ (i.e., vasoreactivity) has been reported to be approximately 10% higher in endurance-trained individuals compared to matching sedentary controls [85], in a way that is modestly (*R*^*2*^ ~ 0.35) related to aerobic capacity [86]. Thus, regular exercise and aerobic fitness may result in a “training effect” on cerebrovascular regulation.

A more in-depth review of cerebrovascular adaptations to hypoxia, high-altitude and physical activity and fitness is beyond the scope of this paper, and we refer the reader to other reviews on these topics [87, 88].

Exploring pathophysiologic alterations in the cerebrovascular function in response to neural injuries can also highlight the importance of this function. On one side of the spectrum are the mild brain injuries (e.g., concussions), which can lead to impaired cerebrovascular function [87–90, 92]. After a mild brain injury, optimal cerebral blood flow is necessary to meet the metabolic needs of the injured brain. However, cerebral blood flow decreases even after a mild injury and can remain reduced for extended periods of time [89, 90]. There may be a neurovascular “uncoupling” [91, 92] and a disruption in cerebral vasoreactivity [93, 94]. In fact, we have recently shown a strong relation between symptom burden and cerebral vasoreactivity after a mild brain injury where higher vasoreactivity was associated with more severe headaches and cognitive symptoms [95]. Moreover, autoregulation may also be impaired with mild brain injuries. For example, one study within 48 h of injury found that almost 30% of patients with mild injuries have impaired or absent cerebral autoregulation [96]. Consistent with these data, in active boxers, autoregulation is impaired due to repetitive, sub-concussive head impact incurred during sparring. These impairments are associated with cerebral hypoperfusion, neurocognitive dysfunction, and marked orthostatic hypotension that manifests beyond the active boxing career [97]. On the other side of the spectrum, in more severe injuries, such as subarachnoid hemorrhage (SAH), there is frequently dysfunction in the cerebrovascular function, particularly in the acute phase [98–100], and this dysfunction appears to be closely related to the clinical and functional outcomes after initial hemorrhage. For example, early impairment in cerebral autoregulation is reported to be a risk factor for delayed cerebral ischemia and subsequent cell death and infarcts [101, 102], as well as for poor acute discharge outcomes [103]. In fact, we have recently found that cerebral autoregulation dysfunction early (within 4 days) after the initial injury is a major factor that contributes to the development of cerebral infarcts and neural cell death [104, 105]. Moreover, the extent of this early dysfunction appears to relate to the rate of functional recovery and overall rehabilitation outcomes months beyond the initial injury: we have recently reported that the extent of cerebral autoregulatory impairment, along with the severity of SAH on admission explains 70–85% of the variation in rehabilitation efficiency and outcome [106]. These data clearly demonstrate the importance of intact cerebrovascular function to mitigate short- and long-term sequelae of both mild and severe brain injuries.

## Conclusions

The data reviewed above show the importance of the cerebrovascular function to support higher neural demand and cognitive function in humans. The evolutionary advantage of higher cognitive function is clear. Yet, even after decades of research, much of the physiology that underlies the main components of the cerebrovascular function—neurovascular coupling, cerebral vasoreactivity, autoregulation—remains only partly understood. Perhaps as a consequence of increased recognition of the link between the cerebrovascular and neural functions, there is a growing interest in understanding physiological underpinnings of the former. There is still much insight into the neural and cognitive functions that could be gleaned from interrogating cerebrovascular function.
